# Anesthesia and evoked responses in neurosurgery

**DOI:** 10.3389/fphar.2014.00074

**Published:** 2014-04-14

**Authors:** Suren Soghomonyan, Kenneth R. Moran, Gurneet S. Sandhu, Sergio D. Bergese

**Affiliations:** ^1^Department of Anesthesiology, Wexner Medical Center, Ohio State UniversityColumbus, OH, USA; ^2^Department of Neurological Surgery, Wexner Medical Center, Ohio State UniversityColumbus, OH, USA

**Keywords:** evoked potentials, brain protection, neurophysiological monitoring, intraoperative monitoring

Intraoperative evoked potential (EP) monitoring has become a routine part of operative neurosurgical procedures. The theoretical, technical, and clinical aspects of various EPs have been extensively characterized and significant clinical experience has been accumulated with this modality of neuromonitoring. Successful EP monitoring requires an adequate understanding of how anesthetic drugs and physiological variations affect EP signals and how to improve the sensitivity of neuromonitoring through appropriate drug selection and administration.

Unlike intraoperative electroencephalography (EEG), EP signals are much smaller in amplitude (0.1–20 mcV) and indistinguishable from background noise. In order to extract the EP signal from the underlying EEG noise, multiple stimulations with summation and frequency filtering are necessary (Freye, 2005; Møller, 2011).

EPs are highly sensitive to fluctuations in physiological parameters such as peripheral and core body temperature, arterial blood pressure, hematocrit etc. They are also susceptible to various general anesthetic agents and other drugs frequently given during surgery.

The effects of general anesthetics on intraoperative EP depend on the mode of evoked response and the pharmacological characteristics of administered anesthetic drugs. Evoked responses that travel via polysynaptic pathways, such as visual EP are significantly more susceptible to the anesthesia and surgery when compared to EPs with fewer synapses in their pathway.

In general, inhalational anesthetics are more potent suppressants of EP than intravenous agents (Banoub et al., 2003; Møller, 2011). Combinations of inhalational agents, as occurs with the addition of nitrous oxide, potentiate the suppressive effects of anesthesia even further.

Despite their suppressive effects on EPs, inhalational anesthetics have obvious advantages for use during neurosurgery because they are easily titratable to provide stable anesthetic conditions. Lower doses of inhalational anesthetics (0.5–0.8 MAC depending on type of the evoked response) have been successfully applied during neurosurgical interventions and neurophysiological monitoring without compromising the quality of monitoring.

Balanced general anesthesia with low doses of inhalational agents combined with low-dose constant infusions of remifentanil (0.05 mcg/kg/min), propofol (50 mcg/kg/min), or dexmedetomidine (0.003–0.005 mcg/kg/min) may be recommended when EP monitoring is anticipated. Such an approach will provide stable anesthesia and reduce the incidence of adverse events encountered occasionally during total intravenous anesthesia such as patient movement and awareness.

Sevoflurane has low solubility compared with other inhalation anesthetics and thus is eliminated rapidly, minimizing its effects during monitoring later in the case (Sloan T, as cited in Fulkerson et al., 2011). Using sevoflurane as an induction agent, Fulkerson and colleagues were able to successfully monitor the intraoperative motor EPs in young children (less than 3 years) undergoing neurosurgical spinal procedures (Fulkerson et al., 2011). We believe that other inhalational anesthetics with low blood solubility (desflurane) may uneventfully be used for anesthesia induction and will be compatible with intraoperative neurophysiological monitoring.

Anesthetics used for intravenous anesthesia, with a few exceptions, produce a dose-dependent suppressive effect on EP. Unlike the other intravenous hypnotics, etomidate, and ketamine tend to increase the SSEP amplitudes (Banoub et al., 2003). Further studies will be required to evaluate whether the use of various concentrations of these anesthetics during neurosurgical interventions suppress EP equally when different modalities of EP are being monitored.

Opioids, in general, do not affect the quality of intraoperative EP monitoring. However, their mild suppressive effects are proportional to lipophilicity. When infused at higher doses, remifentanil causes a 20–80% decline in P37 peak amplitude of SSEP and a mild (<10%) increase in latency (Asouhidou et al., 2010).

Midazolam and other benzodiazepines moderately suppress the intraoperative EP (Banoub et al., 2003), and their use, whenever possible, should be avoided. Benzodiazepine-induced EP suppression is less pronounced compared to inhalational agents.

Dexmedetomidine, a relatively new hypnotic characterized by selective alpha-2 adrenergic antagonism, can be safely used to supplement general anesthesia during EP monitoring (Tobias et al., 2008).

Intravenous lidocaine (1.5 mg/kg/h) is also a useful adjunct to general anesthesia with EP monitoring due to its ability to reduce anesthetic requirements, stabilize the cardiovascular parameters and decrease the incidence of patient movement during surgery (Sloan et al., 2014).

Monitoring of motor EPs during surgery requires special caution, as they are more sensitive to anesthetics and muscle relaxants (Kunisawa et al., 2004; Lotto et al., 2004). Anesthetic conditions optimized for motor EP monitoring are suitable for SSEP registration as well (Pajewski et al., 2007). Although, partial muscle relaxation can be used for motor EP monitoring during surgery (Møller, 2011; Kim et al., 2013), most practitioners refrain from using muscle relaxants after tracheal intubation.

In addition to selection of the most suitable anesthetics, their mode of administration is also an important factor that influences the quality of EP monitoring.

During procedures requiring EP monitoring, steady infusion rates and stable concentrations of inhalational agents are preferred. Administration of drugs in bolus doses and variations in anesthesia level can negatively impact the quality of signal and cause EP suppression indistinguishable from changes triggered by surgical trauma (van Dongen et al., 1999; Lotto et al., 2004; Pajewski et al., 2007; Tobias et al., 2008; Deipolyi et al., 2011).

During lengthy neurosurgical procedures, gradual attenuation of the EP signal may occur. This signal degradation is not related to the dose of anesthetics and is proportional to the length of anesthesia. This phenomenon is more frequently seen in younger patient populations and those with spinal cord pathology (Yang et al., 2012; Macdonald et al., 2013). The exact mechanisms underlying signal degradation are currently not well understood.

Intraoperative monitoring of evoked responses can be successfully utilized to reduce the rate of inadvertent trauma to the nervous structures during neurosurgical procedures. Their interpretation requires profound knowledge of neurophysiology, comprehension of the surgical procedure and an understanding of the effects that general anesthesia and physiological changes may have on signal quality. Intraoperative neuromonitoring is one of the areas of medicine where team approach is a crucial prerequisite to obtain meaningful results.

During neurosurgical procedures, a variety of general and local anesthetics are used, and many of them can substantially affect or even completely eliminate the EP signal. The possibility of anesthesia-related signal suppression and the influence of physiological changes on EP must be considered in order to avoid such effects. Drugs with minimal interference on neurophysiological monitoring should be used preferentially, and attempts made to keep the anesthetic concentrations, temperature, and other physiological variables constant. Maintaining steady state concentrations of an appropriately selected balanced anesthetic will reduce the incidence of false positive results and assist in the prevention of surgical trauma and ischemic damage during neurosurgical interventions. Appropriate drug selection, meticulous drug administration and minimization of physiological variation can improve patient safety by optimizing EP signal monitoring in patients undergoing neurological surgery.

## Conflict of interest statement

The authors declare that the research was conducted in the absence of any commercial or financial relationships that could be construed as a potential conflict of interest.
